# Targeting SHIP1 and SHIP2 in Cancer

**DOI:** 10.3390/cancers13040890

**Published:** 2021-02-20

**Authors:** Chiara Pedicone, Shea T. Meyer, John D. Chisholm, William G. Kerr

**Affiliations:** 1 Department of Microbiology & Immunology, SUNY Upstate Medical University, Syracuse, NY 13210, USA; pediconc@upstate.edu; 2 Chemistry Department, Syracuse University, Syracuse, NY 13210, USA; smeyer@syr.edu (S.T.M.); jdchisho@syr.edu (J.D.C.); 3 Department of Pediatrics, SUNY Upstate Medical University, Syracuse, NY 13210, USA

**Keywords:** SHIP1, SHIP2, cancer, SHIPi, PI3K, AKT, PI(3,4,5)P_3_, PI(3,4)P_2_, Caspase 8, Fas

## Abstract

**Simple Summary:**

Phosphoinositol signaling pathways and their dysregulation have been shown to have a fundamental role in health and disease, respectively. The SH2-containing 5′ inositol phosphatases, SHIP1 and SHIP2, are regulators of the PI3K/AKT pathway that have crucial roles in cancer progression. This review aims to summarize the role of SHIP1 and SHIP2 in cancer signaling and the immune response to cancer, the discovery and use of SHIP inhibitors and agonists as possible cancer therapeutics.

**Abstract:**

Membrane-anchored and soluble inositol phospholipid species are critical mediators of intracellular cell signaling cascades. Alterations in their normal production or degradation are implicated in the pathology of a number of disorders including cancer and pro-inflammatory conditions. The SH2-containing 5′ inositol phosphatases, SHIP1 and SHIP2, play a fundamental role in these processes by depleting PI(3,4,5)P_3_, but also by producing PI(3,4)P_2_ at the inner leaflet of the plasma membrane. With the intent of targeting SHIP1 or SHIP2 selectively, or both paralogs simultaneously, small molecule inhibitors and agonists have been developed and tested in vitro and in vivo over the last decade in various disease models. These studies have shown promising results in various pre-clinical models of disease including cancer and tumor immunotherapy. In this review the potential use of SHIP inhibitors in cancer is discussed with particular attention to the molecular structure, binding site and efficacy of these SHIP inhibitors.

## 1. Overview of Phosphoinositide Signaling

In eukaryotic cells the intracellular leaflet of the plasma membrane lipid bilayer serves as the site where many receptor-based signaling cascades are initiated. Inositol kinases and inositol phosphatases produce and modify inositol phospholipids (PtdIns) at these sites to initiate or in some cases terminate signaling cascades that result in cell survival, proliferation or changes in cellular function [1,2]. These effects are typically the result of changes in gene expression, protein phosphorylation or the inhibition of cytosolic caspases that mediate cell death. PtdIns have acyl chains that anchor them in the inner leaflet of the plasma membrane enabling them to transfer information from membrane-anchored receptors that sense changes in the external milieu of the cell following their engaging ligand [3]. Certain PtdIns species, PI(4,5)P_2_, PI(3,4,5)P_3_, PI(3,4)P_2_, are crucial for initiation of signaling cascades because a diverse array of intracellular kinases, modifying enzymes (e.g., phospholipases, inositol phosphatases) and adapter-scaffold proteins (e.g., Doks, Gabs) are preferentially recruited to them. This recruitment is enabled by pleckstrin homology (PH) and C2 domains present in these proteins that are capable of distinguishing different PtdIns species that have varying numbers of phosphates decorating different carbons in the inositol ring. This chemical diversity at the 6-carbon inositol ring enables a significant degree of information storage and transfer for PtdIns signaling cascades with a theoretical total of 31 PtdIns species that could be generated by inositol kinases and phosphates to trigger different intracellular signaling events. In practice, total information storage content is much less with the phosphate group on the C1 alcohol restricted to simply providing an anchoring linkage to the acyl chains buried in the lipid bilayer, while the C2 alcohol appears not be subjected to phosphorylation likely due to its close proximity to the substituted C1 alcohol and the axial orientation of the C2 hydroxyl on the inositol ring that likely prevents its phosphorylation by intracellular kinases recruited to the inner leaflet. These biological constraints reduce the theoretical maximum to 15 PtdIns species that could be generated at the inner leaflet that can potentially each promote a different intracellular signaling event. However, the genome of vertebrates does not appear to encode the necessary enzymatic and recognition capacity to take advantage of all 15 possible PtdIns species, but investigation of all possibilities is by no means complete. 

**Figure 1:**
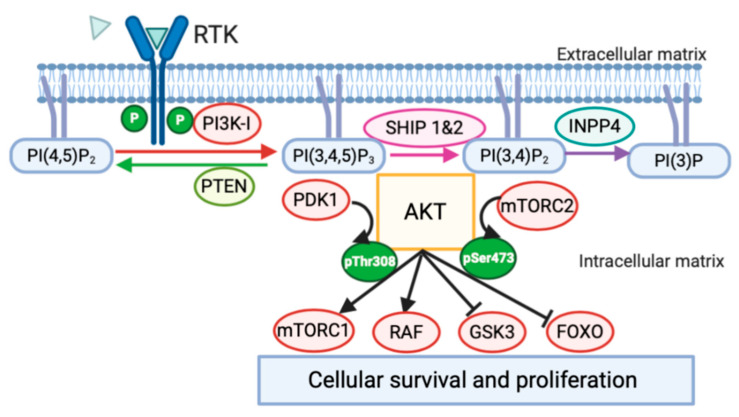
Modification of PtdIns Mediated by PI3K, PTEN, SHIP, INPP4 and the PI3K signaling pathway in cancer: The phosphorylated state of inositol lipids inserted in the cell membrane is controlled by inositol kinase and phosphatase enzymes. As proposed in “The Two PIP hypothesis” [22], the presence of both PI(3,4,5)P_3_ and PI(3,4)P_2_ at the membrane triggers downstream signaling events that more effectively promote cancer cell survival. Downstream targets of AKT are both pro-survival (activation of mTORC1, RAF) and anti-apoptotic (inhibition of GSK-3, FOXO) permitting increased cancer cell survival and proliferation.

Perhaps the most prominent example of PtdIns-mediated information transfer from receptors is the recruitment and activation of the serine-threonine kinase AKT (PKB). AKT contains a PH domain that can recognize both PI(3,4,5)P_3_ and PI(3,4)P_2_, and thus is considered a key distal effector of PI3K signaling [4,5]. After recruitment to these PtdIns species AKT enzyme activity is promoted by its phosphorylation on threonine 308 (Thr308) and serine 473 (Ser473) by other serine-threonine kinases (PDK1, mTORC2) that are also recruited and activated by PtdIns species at the plasma membrane. The PI3K-AKT signaling cascade can impact cell survival, proliferation, effector function and vesicle trafficking [6,7,8,9]. Thus, modulation of inositol kinases and phosphatases that generate or deplete the crucial PtdIns species, PI(3,4,5)P_3_ and PI(3,4)P_2_, by somatic or germline mutation are implicated in many diseases and particularly in cancer [6,10,11,12,13,14,15,16,17,18,19,20,21]. Such enzymes include PI3K, PTEN, SHIP and INPP4 due to their ability to selectively act at the 3′, 4′ and 5′ carbons of the inositol ring, respectively (Figure 1).

## 2. PI3K, The Role of the Different Classes and Isoforms in Cancer Formation and Survival

Phosphoinositide 3 kinase (PI3K), in its various forms, is a key driver of inositol phospholipid signaling pathways, due to its ability place a PO_4_ group on the 3′ hydroxyl of the inositol ring. Three classes of PI3K have been identified in mammals and they consist of heterodimers of a catalytic subunit (p110α, β or γ) and a regulatory SH2 containing subunit (p58α, p55α or p50α). Heterodimers are formed at plasma or intracellular membranes in response to internal or external stimuli that trigger receptor activation. Each PI3K class has a specific cellular localization and can have different PtdIns substrates [8]. Attempts to control the PI3K pathway through inhibition of PI3K were faced with the challenge that isoform specific inhibition was required in some instances [7]. Nonetheless, several isoform-specific PI3K inhibitors have been developed and eventually approved for clinical use in the treatment of cancer [23,24,25,26,27,28], although there are a number of side effects associated with each of these isoform specific inhibitors [28,29]. In addition, the development of resistance to these agents has now been documented in cancer [30]. In order to minimize resistance, these agents are often used in combination with other cancer drugs [31].

Class I PI3K enzymes have been subdivided into subclasses defined by the use of specific regulatory subunits: α, β, δ or γ. While PI3K-α and -β are expressed by all cell types, the PI3K isoforms δ and γ are selectively expressed by lymphocytes. Of these, PI3KCA (containing p110α) over-expression, or activating mutations in it, are known to drive the development of malignancies, as they led to uncontrolled cell proliferation, increased survival, enhance metastasis and inhibition of apoptotic pathways. PI3KCA is upregulated or mutated in multiple human cancers [32,33]. In the PI3KCA class the catalytic subunit is recruited to the cell membrane following activation of a receptor tyrosine-kinase (RTK) or G-protein coupled receptor. Notable RTKs that associate with PI3KCA are the growth factor receptors EGFR and HER2.

After RTK activation the PI3KCA catalytic subunit associates with the regulatory subunit and phosphorylates the substrate PI(4,5)P_2_ forming PI(3,4,5)P_3_ and consuming one molecule of ATP [34]. The presence of PI(3,4,5)P_3_ enables recruitment of select PH domain containing proteins (e.g., AKT) to the plasma membrane. Once recruited to the membrane, AKT is phosphorylated on Thr308 by PDK1 and this leads to activation of the protein kinase activity of AKT [9,35]. Recruitment and phosphorylation of AKT at the plasma membrane triggers cell survival and proliferation through mTORC1 and RAF as well as inhibition of GSK-3 and FOXO signaling [36]. The PI3K/AKT signaling cascade has been shown to be highly relevant in cancer development and survival. Mutations that increase RTK activation [37], PI3K activation [32], and AKT activation [38] are all found to be oncogenic. Furthermore, PI3K class I has an important role in RAS recruitment that leads to triggering of MAP/ERK signaling thus increasing oncogenic potential by promoting cell division [39]. In this signaling cascade the P110α subunit of PI3K interacts with RAS and can also promote angiogenesis through VEGFα signaling [40]. Thus, the therapeutic use of PI3Kα inhibitors in oncology was anticipated to have a profound effect on tumor growth as these inhibitors were expected to lead to rapid cell death. Instead, cells with an inhibited PI3K/AKT pathway seem to enter in a dormant state characterized by nutrient deprivation and decreased neovascularization [41,42]. As these agents do not directly induce rapid cell death, they are often utilized in combination with other antitumor agents. Indeed, there are ongoing clinical trials for PI3Kα inhibitors in combination with AKT and mTORC1 inhibitors and/or standard anticancer therapies with promising results in breast cancer and prostate cancer [43,44]. Notably, combined treatment with the PI3Kα inhibitor Alpelisib and the antiestrogen Fulviestran showed prolonged survival in a recent phase III study [45]. This combination therapy is now FDA approved for treatment of patients with a PI3KCA mutation, hormone receptor positive or HER2 negative breast cancer, as well as in advanced metastatic cancers [23].

Other PI3K isoforms may also be targets for antitumor therapy. A PI3K isoform containing p110β catalytic subunit has oncogenic potential, but seems to be active only in cancers with a PTEN loss of function (LOF) mutation [46]. Phase II clinical trials with selective inhibitors are ongoing in melanomas with this mutant, and are guided by genetic testing to identify patients with such PTEN and PI3Kβ mutations [47]. Importantly, FDA approved PI3K inhibitors active on isoforms δ and/or γ, Duvelisib (δ and γ) [48], Idelalisib (δ) [49] and Copanlisib (α and δ) [50] have demonstrated efficacy in the treatment of hematological malignancies such as chronic lymphocytic leukemia (CLL) and follicular lymphoma (FL). PI3K-δ also has important functions in regulation of the immune system and protection from cancer development [51]. In this case, selective inhibition of a single PI3K isoform is thought to be important. Immunostimulatory activity is associated with PI3Kγ inhibitors which can promote polarization of tumor associated macrophages from anti-inflammatory (M2) to inflammatory (M1) inducing cytokine production to promote an anti-tumor immune response [52]. PI3Kδ also plays a role in B and T cell receptor signaling, thus specific inhibitors for this PI3K isoform are used in T and B cell malignancies where tonic signals from the BCR and TCR contribute to cancer cell survival, but also inhibit survival of tumor-associated regulatory T cells (Treg) [51]. On this basis ongoing clinical trials featuring PI3K inhibitors α and δ as combined chemotherapeutics and immunotherapeutics for solid tumors are being pursued [53]. 

PI3K class II adds a phosphate at the 3′ hydroxyl of PtdIns and PI(4)P leading respectively to P(3)P and PI(3,4)P_2_ production. This PI3K class is recruited in the phagosome, endosome and lysosome where it helps regulate endocytosis, receptor recycling, membrane trafficking, and clathrin mediated phagocytosis essential for nutrient uptake [54,55]. The importance of this PI3K class is exemplified by its three distinct isoforms: PI3K-C2α that regulates vesicle trafficking, PI3K-C2β involved in cell migration, PI3K-C2γ that controls AKT2 activation [56]. Notably PI3K-C2β has shown importance in invasiveness of prostatic cancer via the MEK/ERK pathway [57].

PI3K class III (also called VPS34) contributes to the PI(3)P pool by phosphorylating PtdIns on the 3′ position. This PI3K class III regulates autophagy and phagocytosis in both normal and transformed cells [58]. There are studies correlating class II and III PI3Ks to cancer growth via an alternative signaling pathway that is proposed to be independent of AKT activation. An example is VPS34-dependent enrichment of PI(3)P on an endosomal membrane promoting SGK-3 recruitment [58]. Once SGK-3 is recruited, it is then phosphorylated by PDK-1 and mTORC2 at the vesicular membrane which then permits mTORC1 activation [59]. This protein kinase signaling pathway resembles the PI3K/AKT axis and is upregulated in breast cancer cells as a survival strategy after prolonged PI3K/AKT inhibitor treatment—and thus constitutes a mode of PI3K inhibitor resistance [59]. PI(3,4)P_2_ and PI(3)P are also products of phosphatase activity from other more phosphorylated forms of PtdIns (e.g., PI(3,4,5)P_3_). For this reason, not only kinases, but also inositol phosphatases can have an important impact on shaping cellular signaling in health and disease. 

## 3. Phosphatases Are Also Important Modulators of PtdIns

Inositol phosphatases influence PI(3,4,5)P_3_ levels in cells by controlling the rate of phosphate hydrolysis at a specific position of the inositol ring. Although the PI3K product PI(3,4,5)P_3_ is well known to be a potent activator of cancer cell survival by promoting AKT activation, PI(3,4)P_2_ can also bind to the PH domain of AKT, and in fact does so in a manner that leads to more potent AKT activation through phosphorylation of AKT on Ser473 [4,60]. As put forward in the “Two PIP Hypothesis”, the presence of both PI(3,4,5)P_3_ and PI(3,4)P_2_ promote dual phosphorylation of AKT on Thr308 (by PDK1) and Ser473 (by mTORC2) and consequently a greater degree of AKT activation at the plasma membrane [35]. Thus, the presence of both PI(3,4,5)P_3_ and PI(3,4)P_2_ appears to be important for the survival of cancer cells as shown in studies where exogenous PI(3,4)P_2_ addition to cancer cells can protect them from cell death induced by chemical inhibitors of the 5′ SH2-containing inositol phosphatases that hydrolyze PI(3,4,5)P_3_ to PI(3,4)P_2_ [19,61].

The cellular concentration of the PI3K product PI(3,4,5)P_3_ is modulated by two different types of inositol phosphatases in the cell: PTEN (phosphatase and tensin homolog protein) and the Src homology 2 (SH2) domain containing inositol polyphosphate 5′phosphatases, SHIP1 and SHIP2, here collectively referred to as SHIP [62,63]. Although PTEN and SHIP activity decrease PI(3,4,5)P_3_ concentration, they give rise to different products with very different signaling potentials. PTEN converts PI(3,4,5)P_3_ to PI(4,5)P_2_, while SHIP converts PI(3,4,5)P_3_ to PI(3,4)P_2_ (Figure 1) [62]. PI(4,5)P_2_ and PI(3,4)P_2_, can then be further converted to PI(4)P and PI(3) by the PI(4,5)P_2_ 5′ phosphatase and INPP4A/B P, respectively. Because their PtdIns substrates promote cell survival and growth. PTEN [64,65,66], PI(4,5)P_2_ 5′ phosphatase [67] and the INPP4A/B genes [68,69] also function as tumor suppressors. In fact, inactivating PTEN mutations are frequently present in tumors [70]. Such mutations alter PTEN localization, catalytic activity or protein levels and are most frequently found in Non-Small Cell Lung Cancer (NSCLC) and triple negative breast cancer [65]. However, PTEN has both phosphatase-dependent and phosphatase-independent activities. The latter can be nuclear, through chromatin remodeling stability and dsDNA break repairs, but also cytoplasmatic by inhibiting FAK or Shc. Although PTEN is a haploinsufficient tumor suppressor, loss of heterozygosity (LOH) or decreased gene dosage seems to drive tumorigenesis [70,71,72]. There are currently no strategies for restoration of PTEN activity in cancer; however, patients with cancers that have PTEN LOF mutations are selectively being treated in clinical trials with PI3K inhibitors (to PI3Kα or β) to limit the PI3K/AKT signaling pathway that has become unrestrained due to excess PI(3,4,5)P_3_ production following reduced PTEN activity [66].

The 4′ phosphatase INPP4 depletes AKT-activating PI(3,4)P_2_ from the plasma membrane by converting it to the inactive monophosphate PI(3)P. Two INPP4 enzymes are present in the human genome: INPP4A and INPP4B. LOF mutations in the latter are correlated with colon cancer [68], ovarian cancer [73] and breast cancer [74] through activation of the PI3K/AKT/SGK pathway [69]. INPP4B can also dephosphorylate PI(3,4,5)P_3_ that accumulates in cancers with PTEN deficiency leading to more aggressive cancers [75,76]. In fact, association of PTEN LOF with an INPP4B inactivating mutation are present in triple negative breast cancer that have a particularly poor prognosis [77]. Conversely, there is a study that suggests the oncogenic potential of INPP4B in a subset of melanomas. In this setting INPP4B is overexpressed and activates cell proliferation through the GSK3/MAPK pathway suggesting that this mechanism is independent of the PI3K/AKT pathway and probably cell-specific [78]. As for PTEN, no treatment strategies specifically targeting INPP4B function are currently available, and thus PI3K or its distal effectors might also be targeted in cancers where INPP4B function is altered.

## 4. The Dual Role of SHIP in the PI3K/AKT Cascade Regulation 

**Figure 2:**
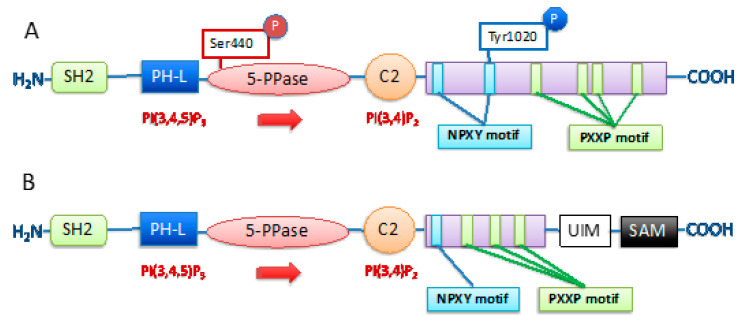
SHIP protein structure and active sites SHIP1 and SHIP2 are multidomain proteins constituted of: SH2 domain, PH-L domain, 5′-phosphatase catalytic domain, C2 domain and C-terminal domain containing PXXP and NPXY motifs. (**A**) SHIP1 has phosphorylation sites on Serine 440 (Ser440) and Tyrosine 1020 (Tyr1020) that regulate respectively its activation and ubiquitination. (**B**) SHIP2 has a ubiquitin interacting motif (UIM) and a sterile alpha motif (SAM) domain, not present in SHIP1.

SHIP has a central role in PI3K signaling pathway by converting PI(3,4,5)P_3_ to PI(3,4)P_2_. There are two SHIP paralogs associated with the PI3K/AKT pathway: SHIP1 and SHIP2. In general, these two enzymes have a high homology with significant sequence divergence in their SH2 domains and the presence of a SAM domain and a ubiquitin interacting motif in SHIP2 (Figure 2) [79,80,81,82], but they show significant difference in their expression profile. SHIP1 is expressed by all cells of the hematolymphoid lineages [83], including embryonically-derived brain-resident microglia [84], mesenchymal stem cells and their osteoblast progeny [83,85], and thus has a key role in immune system regulation, bone formation and stem cell function [80,81,83,85,86,87,88]. SHIP2 is expressed by all cells in the body [89] and can act as a negative regulator of insulin signaling [90,91]. Hence, their differing roles in promoting or inhibiting PI3K signaling in a given cell at a given time is determined by a combination of different factors, including expression, specificity of recruitment to various signaling complexes at the plasma membrane and binding kinetics of their respective SH2 domains for phosphorylated receptor tails [92]. SHIP protein levels are regulated in many ways, including by transcriptional, post-transcriptional and post-translational mechanisms [22,93]. SHIP1 phosphatase activity is increased by phosphorylation on Ser440 by PKA [94], while its phosphorylation on Tyr1021 appears to induce its ubiquitination and proteasomal degradation [95] (Figure 2). Post transcriptional regulation of INPP5D (SHIP1) with MicroRNA-155 (miR-155) has shown to impact tumor progression [96]. MiR-155 upregulation is associated with poorer prognosis in B cell lymphoma [97,98], although upregulation of miR-155 in dendritic cells improves their maturation and promotes adaptive immune response by CD8+T cells [99]. MicroRNA targeting of INPPL1 (SHIP2) by miR-205 has recently been correlated with oncogenic transformation in melanoma [100]. However, both SHIP paralogs may enhance AKT activation through production of PI(3,4)P_2_ as this PtdIns promotes AKT activation more effectively than the SHIP1/2 substrate (PI(3,4,5)P_3_) (Figure 1) [5]. As proposed in “The Two PIP Hypothesis”, this AKT-activation induced by PI(3,4)P_2,_ a SHIP1/2 product, is especially relevant for cancer cell survival [19,22,61], but also interestingly for CMV infected cells [101]. Thus, through their capacity to reduce PI(3,4,5)P_3_ while producing PI(3,4)P_2_ at the plasma membrane, both SHIP1 and SHIP2 can promote cancer cell survival, metastasis and growth [19,22,102]. 

## 5. Molecular Basis for Targeting SHIP1 or SHIP2 in Cancer 

Below we summarize the current evidence in support of targeting SHIP1 and/or SHIP2 to limit cancer cell survival, metastasis or promote its immune control.

### 5.1. SHIP1 and SHIP2 Produce PI(3,4)P_2_ to Increase AKT Activation and Cancer Cell Survival

As discussed above SHIP1 and SHIP2 convert the PI3K product PI(3,4,5)P_3_ to PI(3,4)P_2_ to create a mixture of both PtdIns species at the plasma membrane. Both of these PtdIns promote activation of AKT due to the ability of its PH domain to interact with both PI(3,4,5)P_3_ to PI(3,4)P_2_ with differing affinity [4,5]. Presence of both PtdIns may be anticipated to further increase activation of AKT to help tumor cells avoid intrinsic cell death mechanisms [4,60]. In support of this hypothesis, increased levels of PI(3,4)P_2_ have been observed in leukemia cells [103]. Increased levels of PI(3,4)P_2_ due to mutations in INPP4A/B genes also promote the transformation and tumorigenicity of mouse embryonic fibroblasts (MEF) and breast tumor formation [76,82,104,105]. Studies from our groups have established that PI(3,4)P_2_ directly promotes increased AKT activation and survival in a number of cancer cell types as introduction of exogenous PI(3,4)P_2_ into these cancer cells protects them from cell death induced by SHIP1-selective or pan-SHIP1/2 inhibitors [19,61]. In fact, the distinctive ability of SHIP inhibitors to limit PI(3,4)P_2_ at the plasma membrane could be advantageous for treating tumors that are resistant to AKT and/or mTOR inhibitors, as they may still be susceptible to SHIP inhibition. This pathway may be particularly germane for epithelial cancers that express and utilize EGF-R, as SHIP2 has been shown to be recruited to EGF-R where it can promote a multitude of signaling pathways to facilitate carcinogenesis, including AKT activation, CXCR4 expression and cell migration [106,107]. Hyperactivation of EGFR-R and its signaling cascade are known to increase cancer survival and aggressivity [107]. Interestingly, in absence of SHIP2, EGFR is internalized and degraded and fails to promote cell signaling [108]. Thus, SHIP2 could be a cancer target independent of its ability to promote AKT activation by PI(3,4)P_2_ production. Furthermore, PI(3,4)P_2_ can promote mTORC1 activity and cell growth when produced at the plasma membrane, while it can inhibit mTORC1 activity when generated on lysosomal membranes in nutrient-deprived cells [109]. It is also worth noting that while both AKT and mTOR can now be targeted directly in cancer with small molecules, resistance to these therapies can arise. Thus, SHIP inhibition offers a novel and alternative means to target PI3K pathways that drive cancer cell survival.

### 5.2. SHIP1 Sets a Threshold for Cell-Extrinsic Apoptosis

Tumor cells frequently avoid cell death via increased PI3K-AKT signaling that overrides cell-intrinsic cell death mechanisms. However, CD95/Fas mediated cell-extrinsic cell death is another potential means for the body to control cancer cell growth, and particularly through the T cells that can express the ligand for Fas, FasL. In vitro studies indicated SHIP1 could oppose cell-extrinsic cell death in T cells by impairing CD95/Fas glycosylation, independent of its enzymatic function [110]. How SHIP1 achieved this mechanistically was not determined and the physiologically relevance of this was not clear. Subsequently, it was found that SHIP1 is physically recruited to CD95/Fas to impair its capacity to activate the Caspase required for the extrinsic cell death pathway, Caspase 8 [111]. This function of SHIP1 does appear to require enzymatic activity as a SHIP1-selective inhibitor, 3AC, promotes increased Caspase 8 activation and cell death downstream of CD95/Fas in both murine and human T and B lymphoma cells [111]. This molecular function of SHIP1 is also important for survival of T cells at mucosal surfaces, lungs and intestine, as administration of a Caspase 8 inhibitor to SHIP1^−/−^ mice selectively rescued T cell survival in these tissues and consequently reduced myeloid-mediated lung and gut inflammation that compromises viability in SHIP1^−/−^ mice [111]. Thus, targeting of SHIP1 in hematolymphoid cells that express SHIP1, and have an intact Fas-Caspase 8 cell death pathway, is also a therapeutic option for such cancers. Whether this might also be possible for epithelial cancers, that do not express SHIP1, awaits analysis of whether SHIP2 has a similar role in limiting Fas signaling in epithelial tumor types. 

### 5.3. Selective Targeting of SHIP1 to Enhance Immune Control of Cancer

SHIP1 has an important role in regulation of both innate and adaptive immune responses [22]. However, genetic mutation of SHIP1 in a germline, inducible or NK cell-specific fashion was found to promote increased numbers of MDSC and Treg cells or decreased NK cell function—none of which are consistent with the use of SHIPi to promote tumor immunity [112,113,114,115,116]. Consistent with these genetic analyses, extended treatment of mice with a SHIP1-selective inhibitor recapitulated these phenotypes, including increasing MDSC numbers, reduced priming of T cell responses, increased Treg numbers and a hyporesponsive NK compartment [61,117]. We reasoned that extended genetic SHIP1 deficiency or chemical inactivation leads to over-stimulation of tumor-responsive T and NK cells that then disables them—referred to as ‘disarming’ in the NK cell field. This appears to be the case as by simply switching from extended, daily administration of the SHIP1 inhibitor, to a pulsatile dosing strategy that allows them to recover from stimulation, improved immune control in three different tumor models [118]. This effect was correlated with increased responsiveness of both NK cells and T cells through major activating receptors, including the TcR, NKp46, NKG2D. In fact, improved tumor control by SHIPi was found to be dependent upon both host NK and T cell function [118]. The impact of pulsatile SHIPi treatment on tumor antigen presentation by APC and on the immune microenvironment in tumors has yet to be explored. However, studies of SHIP1 KO mice and/or pulsatile SHIPi treated mice suggests there is also the potential to improve dendritic cell function in cancer [99,113] and bias the tumor macrophage compartment toward more inflammatory and phagocytic cells to further facilitate immune control [119,120,121]. The latter is a particularly intriguing notion for glioblastoma tumors as these brain tumors have a large microglial component whose phagocytic functions can be enhanced by SHIPi compounds that can penetrate the CNS [84]. 

### 5.4. SHIP2 Enhances Cell Migration, Invasion and Metastasis

SHIP2 has been found to be over-expressed in variety of cancers, including breast, colon, and glioma where it is associated with poor prognosis [102,122,123]. In these epithelial-derived tumors the SH2-containing SHIP1 isoforms are not thought to be expressed; however, an internal promoter is active in cancer stem cell populations present in epithelial tumors suggesting the s-SHIP stem cell-specific isoform of SHIP1 [124] may be expressed and contribute to tumor stem cell survival [125,126,127]. The more aggressive prognosis of these epithelial cancers that overexpress SHIP2 may be due to increased cell migration and invadopodia maturation [128], leading to increased metastatic capacity [100,123]. Conversely, SHIP2 has been reported to regulate focal adhesion and suppress motility of PTEN-deficient glioblastoma acting on PI(4,5)P_2_, at the plasma membrane [129] but SHIP2 inhibition showed reduced fibronectin-dependent cell migration in glioblastoma [130,131]. Interestingly, SHIP2 has been reported to be present in nuclear speckles when phosphorylated on Serine 132 [132]. Thus, nuclear localization of SHIP2 may regulate PtdIns composition in the nuclear membrane and ultimately lead to chromatin remodeling and transcriptional regulation that have been correlated to epithelial to mesenchymal transition [133,134]. Furthermore, PI(4,5)P_2_ can also be found trapped by nuclear proteins in speckles and have an important role in regulate pre-mRNA splicing [135,136]. SHIP2 has also been reported to contribute to podosomes and invadopodia formation through its product PI(3,4)P_2_ [137]. These actin-rich structures containing matrix peptidases and metalloproteases have a fundamental role in cancer migration and extracellular matrix degradation [138]. SHIP2 promotes invadopodia maturation through PI(3,4)P_2_ accumulation at the growth cone initiated by the tyrosine kinase Tks5 in breast carcinoma [128]. As discussed above, SHIP2 through its’ production of PI(3,4)P_2_ may simply promote resistance to cell death by increasing activation of AKT. Additionally, SHIP-deficient mice and mice treated with a SHIP1 selective inhibitor (3AC) have lower levels of SDF-1/CXCL12 in secondary lymphoid organs, bone marrow and plasma [117,139]. SDF-1/CXCL12 is a chemokine used by metastatic cells and cancer stem cells to disseminate and colonize a new niche. Thus a potential use of SHIP1 selective inhibitors in epithelial cancers that lack it expression could be to impair homing of metastatic cells by tumor niche cells and in doing so increase the life expectation of stage 4 metastatic cancer patients [140]. 

## 6. Comparison of Crystal Structures of the SHIP1 and SHIP2 Phosphatase Domains

**Figure 3:**
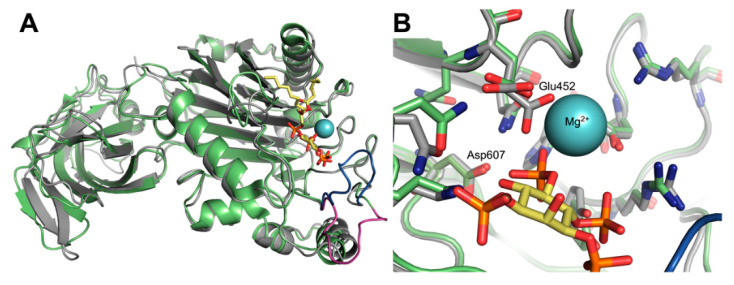
Overlap of X-ray structures of SHIP1 and SHIP2 containing C2 domain and phosphatase domain. (**A**) Structure of SHIP1 (in grey, 6IBD) and SHIP2 (in green, 5OKM) aligned with a model of PI(3,4,5)P_3_ constructed as described by Lietha [144], where coordinates for the inositol and metal ion are taken from PDB: 3MTC [146]. The C2 domain is on the left and the phosphatase domain is on the right. In the SHIP1 crystal the P4IM flexible loop region is in the “in” conformation (in blue), while in the SHIP2 crystal structure this portion of the enzyme is in the “out” position (in violet). (**B**) Closeup of the 5′-phosphate binding site from (**A**), SHIP1 in grey, SHIP2 in green. The residues in SHIP1 and SHIP2 have close homology in both structure and position. Some disorder is seen with Glu452 in the SHIP1 structure 6IBD, two conformations are given in the structure, but Glu473 in 5OKM overlaps with one of the conformers and likely can access the other with little difficulty.

More detailed structural information about SHIP has now become significantly more abundant, with several groups recently reporting x-ray crystal structures of the phosphatase domain of SHIP1 [141,142]. To date the full length protein of SHIP1 or SHIP2 has not yielded to crystallization, however crystals have been generated from sections of the protein. At first these structures focused on the SHIP2 phosphatase domain [143], but more recently these structures include both the catalytically active phosphatase domain attached to the neighboring C2 domain for SHIP2 [144] and SHIP1 [141,142]. The inclusion of the C2 domain is important, as this section of the enzyme includes the site where PI(3,4)P_2_ and agonists have been shown to interact with the enzyme to enhance its phosphatase activity [145]. These structures allow for a direct comparison of the similarity of the enzymes in a three dimensional matrix. SHIP1 and SHIP2 have been reported to be ~60% similar in amino acid sequence, and therefore are predicted to have very similar structures. The x-ray structural data are consistent with this prediction, for example the two crystal structures containing the phosphatase and C2 domains of SHIP2 and SHIP1 (5OKM [144] and 6IBD [141] respectively) show high structural similarity (RMSD = 0.641 Å (2369 to 2369 atoms) (Figure 3). Most of the key elements (β-sheets and α-helical substructures) occupy overlapping regions in the two structures. Some differences are seen in the flexible loop regions, especially in the P4-interactive motif (P4IM) loop region (blue and violet, Figure 3A). The active site of the phosphatase domain is very similar, with most secondary and tertiary structure demonstrating high homology between SHIP1 and SHIP2 (Figure 3B). The key aspartic acid that cleaves the 5′ phosphate (Asp607 in SHIP1, Asp586 in SHIP2) are in virtually identical positions in the two structures (Figure 3B). Some amino acids show some disorder and multiple possible conformations (like Glu452 in the SHIP1 structure, which interacts with the magnesium ion which acts as a Lewis acid in the active site), but this is not unusual and the differences are minimal.

While the phosphate binding site of the phosphatase domain of SHIP1 and SHIP2 are very similar, some other areas of the enzyme show significant differences. The region closest to the inositol binding site that is most different is the P4IM flexible loop region which interacts with the 3′ and 4′ phosphate [143]. This flexible loop can be seen in Figure 3A, where the loop is in violet for the SHIP2 structure (PDB: 5OKM) and in blue for the SHIP1 structure (PDB: 6IBD). Potter and co-workers describe molecular dynamics studies where this loop can rest in an open conformation (as seen in the SHIP2 structure in Figure 3A) or a closed conformation, which has significantly more interactions with the 3′ and 4′ phosphates of PI(3,4,5)P_3_ [143]. In the overlapping structures in Figure 3 both conformations have been captured, although given the flexibility of the loop the conformations should probably be considered a snapshot of a mobile section of the protein. 

**Figure 4:**
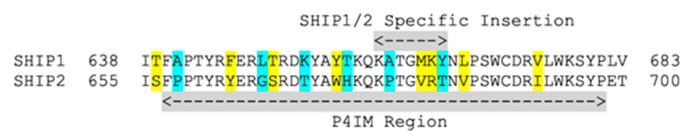
Sequence comparison of the SHIP1 and SHIP2 P4IM region [146]. Conservative amino acid differences are highlighted in yellow, radical amino acid differences are highlighted in blue.

Analysis of the amino acid sequence of the P4IM region in SHIP1 and SHIP2 reveal significant differences between SHIP and other 5′-inositol phosphatases. The two SHIP paralogs have a longer P4IM loop than other 5′-inositol phosphatases, with an insertion of seven amino acids that is not present in the other enzymes (Figure 4) [146]. The entire P4IM region is ~40 amino acids in length, with 13 of these amino acids being different between the SHIP paralogs. The SHIP2 P4IM loop is significantly more rigid than that the SHIP1 loop, having five prolines as opposed to only three prolines in the SHIP1. The greatest area of difference appears to be near the SHIP1/2 specific insertion region. This area of the protein appears ripe for a mutagenesis study to determine which amino acids are key in determining selective inhibition of SHIP1 and SHIP2, as some selective inhibitors of either enzyme have been disclosed (the aminosteroid 3AC 9 is a selective SHIP1 inhibitor [61] and AS1949490 **7** is a selective SHIP2 inhibitor [147]). This may include swapping the entire loop sequence between SHIP1 and SHIP2, just the area near the SHIP specific insertion or point mutations to determine which amino acids are key in providing paralog selective inhibition.

Mui and co-workers have reported the identification of the site on the C2 domain of SHIP1 where PI(3,4)P_2_ and SHIP1 agonists bind to accelerate the phosphatase reaction [148]. The identification of this site should accelerate the development of SHIP1 agonists, which also may exhibit antitumor properties [145]. Investigations by Leitha and co-workers into the mechanism of allosteric communication between the C2 domain and the phosphatase domain in SHIP2 using molecular dynamics simulations and amino acid mutagenesis implicated conformational changes in three loop regions as leading to the acceleration of the phosphatase activity [144]. Overall, this study delineated changes in protein dynamics and active site stabilization rather than large conformational changes as the main causes of the increase in SHIP2 phosphatase activity. The high overall sequence identity that SHIP1 shares with SHIP2 (~50%) suggests that the mechanism of allosteric acceleration may be similar between the enzymes [149].

## 7. Mechanism of SHIP1 and SHIP2 Catalysis

The mechanism of the catalytic phosphate hydrolysis of PI(3,4,5)P_3_ by SHIP has been intensively investigated. The first mechanism for the catalysis by 5′-inositol phosphatases like SHIP was proposed by Mitchell and co-workers, who used the homology between the inositol phosphatases and AP endonucleases to guide their studies [150,151]. This work identified six conserved motifs in the 5-phosphatase family and, using site-directed mutagenesis, validated that these conserved motifs are critical for catalytic activity. These residues included a glutamic acid residue which is important for binding of a Mg^2+^ ion in the phosphatase active site. This metal ion serves as a Lewis acid to activate the 5′-phosphate for hydrolysis. Several asparagines and a histidine were also highly conserved, with these sidechains being utilized to bind to the phosphates on the inositol. A key aspartic acid (Asp586 SHIP2, Asp607 SHIP1) was identified as being involved in the hydrolysis of the phosphate, which directs addition of a water molecule to the 5′-phosphate as proposed in the mechanism of AP endonuclease [152]. The addition of the water molecule to the phosphate then was proposed to lead to a transient pentavalent phosphate intermediate, which then proceeds to the observed products of inositol and free phosphate. Phosphatases may hydrolyze phosphates through associative or dissociative mechanisms [153], but an associative mechanism is supported by both O^18^ labeling [154,155] and molecular dynamics simulations [156] for AP endonucleases. Given the strong sequence similarity between the inositol phosphatases and the AP endonuclease it is very likely that the mechanisms proceed through similar pathways. Still, questions remained about the number of metal ions in the transition state, the movement of the metal ion during phosphate cleavage and the orientation of the inositol in the active site [157].

**Figure 5:**
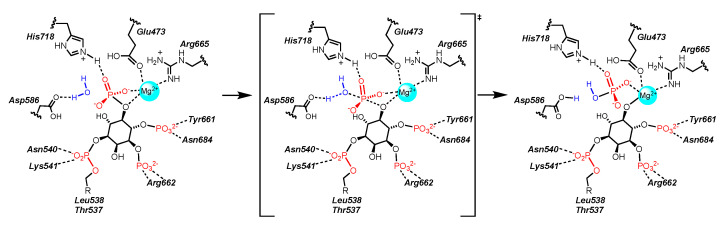
Catalysis of the cleavage of the 5′-phosphate of PI(3,4,5)P_3_ by SHIP2.

The recent crystal structures of the phosphatase domains of SHIP2 [143,144,146] have led to a greater understanding of the number of metal ions in the active site, the specific residues that are interact with the substrate, and the orientation of the inositol during the hydrolysis of the 5′-phosphate. The mechanism that emerges is summarized in Figure 5 below using the SHIP2 residues [158]. After binding in the active site, a magnesium ion directed by a nearby glutamate coordinates to the 5′-phosphate, activating the phosphate to nucleophilic attack. The inositol is held in place by interactions between a number of other residues and the phosphates on the inositol ring. Overall, the PI(3,4,5)P_3_ binding site it quite polar, with many basic and acidic residues. A key aspartic acid residue (Asp586 in SHIP2, Asp607 in SHIP1) then directs a water molecule to interact with the 5′ phosphate, facilitating the hydrolysis. The protonation of magnesium salt of the inositol has not been studied in detail, but several polar sidechains (like the nearby Glu473) may also facilitate a proton transfer to liberate the PI(3,4)P_2_ product of the enzyme.

## 8. Identification and Development of SHIP1 and SHIP2 Inhibitors

Given the strong interest in the role of SHIP1 and SHIP2 in many disease states (including such important disease areas as cancer, obesity and Alzheimer’s disease) [159], several studies have described small molecules that can modulate the phosphatase activity of SHIP1 and/or SHIP2. The identification of small molecule SHIP inhibitors (SHIPi) through screening campaigns are often complicated by the significant homology of the 5′-inositol phosphatases, which can lead to problems with enzyme selectivity [160]. Still, some successes were documented in these areas. These small molecules can now be utilized to gain a better understanding of the underlying biological processes the SHIP enzyme influences. Additionally, these agents can be used in animal studies to determine what type of SHIP inhibitor (selective SHIP1 [61], selective SHIP2 [147], or pan-SHIP1/2 inhibitors [84]) are best suited for use to improve the specific condition. Below we describe recent developments in SHIPi, as several reviews already encompass earlier work in this area [161,162].

**Figure 6:**
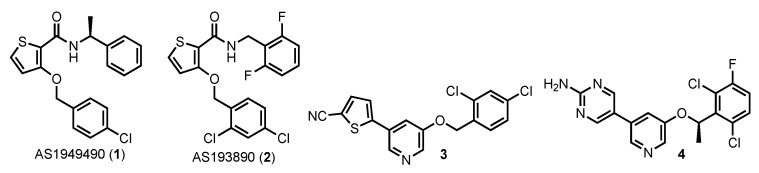
SHIP2 Inhibitors.

Perhaps the best known SHIP2 inhibitors were reported by Suwa and co-workers at Astellas Pharmaceuticals (Tokyo, Japan). Initially AS1949490 (7) was disclosed (Figure 6), which showed selective inhibition of SHIP2 (IC_50_ of 0.62 µM, against SHIP1 the IC_50_ was 13 µM) in the malachite green phosphatase assay using Ins(1,3,4,5)P_4_ as the substrate [147]. No inhibition of other inositol phosphatases (PTEN, synaptojanin and myotubularin) was observed at concentrations up to 50 µM. A kinetic analysis of SHIP2 and AS1949490 1 showed that the inhibition was competitive with the substrate, meaning that the inhibitor was binding at the active site of the phosphatase. AS1949490 was found to increase glucose consumption in L6 myotubes and increase gluconeogenesis in cultured hepatocyte FAO cells. Studies in *db/db* mice demonstrated that the compound significantly reduced plasma glucose levels without effecting body weight, insulin levels or food intake. These results are some of the strongest that support the use of SHIP2 inhibitors to treat diabetes. Further studies by the Astellas researchers disclosed that AS193890 (2) was also a potent inhibitor of SHIP2 [163], with similar potency and selectivity to AS1949490. This compound also showed significant activity in cell-based assays. AS1949490 (7) has been utilized to probe the role of SHIP2 in a number of systems [164,165,166,167]. Relevant to this review, SHIP2 has been postulated to play a role in development of breast cancer [106,107,122,168], and recent studies with AS1949490 (7) provide evidence that SHIP2 is involved in breast cancer metastasis [131,169]. While these studies portend a role for SHIP2 inhibitors in breast cancer treatment, the thiophenes 1 and 2 are generally regarded to have poor pharmacodynamic properties [143], which is common in small molecule leads but precludes clinical development. This may be due to oxidation of the thiophene ring by cytochrome P450 enzymes [170,171]. Analogs with better PK/PD profiles would have to be developed, likely by removing the thiophene.

More recently a series of pyridine based SHIP2 inhibitors based on crizotinib was disclosed as having SHIP2 inhibitory activity [172], with the most potent compounds being thiophene 3 (IC_50_ of 3.2 µM vs. SHIP2) and the aminopyrimidine 4 (IC_50_ of 2.0 µM vs. SHIP2). While no data on the inhibition of other 5′-inositol phosphatases was provided, both 3 and 4 showed low toxicity against HT22 cells, an immortalized mouse hippocampal neuronal cell line. Interestingly, both crizotinib and AS1949490 showed much greater toxicity when tested on this cell line. The aminopyrimidine 4 also showed good stability against degradation by a small panel of cytochrome P450 enzymes and was more inert than 3 in a liver microsome degradation assay. Initial pharmacokinetics were also presented on aminopyrimidine 4, with the compound demonstrating favorable properties including good oral bioavailability. The compound can now be considered a candidate for in vivo efficacy studies to evaluate the potential of SHIP2 inhibitors as therapeutics for Alzheimer’s disease, with the caveat that other 5′-inositol phosphatases may also be affected and SHIP1 was recently shown to be a potential target for Alzheimer’s disease therapies [84].

**Figure 7:**
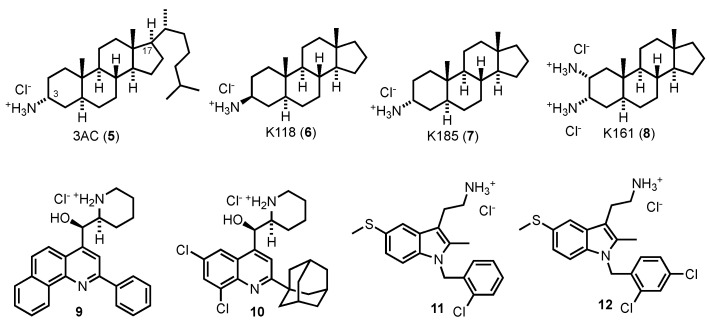
Aminosteroids, quinoline and tryptamine SHIP antagonists from Kerr and co-workers.

A number of other SHIP inhibitors been found from high throughput screening performed by the Kerr group at SUNY Upstate Medical University (Figure 7). The first inhibitor that was disclosed from these studies was 3α-aminocholestane (3AC) 9, which was shown to be a selective inhibitor of SHIP1 with a potency of ~10 µM [61]. Consistent with the SHIP1 inhibition, the molecule was shown to boost granulocyte production, increase immunoregulatory capacity and enhance blood cell recovery in myelosuppressed hosts [61]. Additionally, transient inhibition of SHIP1 did not lead to the lung pathology and pneumonia observed in SHIP1 knockout mice. This molecule also decreased the growth and survival of leukemia (KG-1 and C1498 cells) and multiple myeloma cells, but not osteosarcoma cells that lack SHIP1 expression. Further control experiments showed that these cytotoxic effects could be ameliorated by adding the product of the SHIP1 reaction, PI(3,4)P_2_ to the growth media while the addition of another inositol bis-phosphate, PI(3,5)P_2_, did not show this effect [61]. The antitumor effects of 3AC were robust enough to be observed in a xenograft challenge in mice with OPM2 cells [19], where 3AC was shown to significantly enhance survival of myeloma-engrafted mice after tumor challenge. Interestingly, 3AC treatment de-stabilizes SHIP1 protein expression via the Ubiquitin-proteasome system, but not SHIP2, in multiple myeloma cells, providing further evidence of its selectivity [19]. One drawback with 3AC is the poor water solubility of this scaffold, which requires a special protocol for in vivo dosing and solubilization in ethanol for in vitro studies [61]. This led us to develop a number of aminosteroid analogs of 3AC with increased solubility through eliminating the C17 alkyl sidechain or by adding polar groups to the steroidal ring structure. To date we have characterized several such derivatives biologically including K118 (6) [88], K185 (7) and K161 (8) [84]. Interestingly, all three molecules show significant inhibition of SHIP2 as well as SHIP1, implicating a role for the substituent at the C17 position in controlling selectivity for SHIP1 vs. SHIP2.

In addition to aminosteroids some other scaffolds were discovered by the Kerr group during their high throughput screening campaign. These include the quinoline amino alcohols 9 and 10, which are derived from a program at Walter Reed on the development of new antimalarials [173], which culminated in the discovery of mefloquine [174]. Both quinolines 9 and 10 were pan-SHIP1/2 inhibitors, and induced apoptosis in the OPM2 multiple myeloma cell line [19]. Interestingly, mefloquine was also shown to inhibit both SHIP1 and SHIP2 (albeit at significantly higher concentrations than it is used as an antimalarial) [175] which may explain the antitumor properties observed for mefloquine at higher concentrations [176]. An initial synthetic exploration of the quinoline structures determined that the piperidine ring and alcohol were not necessary for SHIP inhibition, with simplified quinoline-amine structures also demonstrating significant SHIP inhibition in the malachite green assay [175]. These quinoline pan-SHIP1/2 inhibitors also showed cytotoxicity against a SHIP2 expressing breast cancer cell lines [19], including MCF-7 and triple negative MDA-MB-231 cells. 

A third class of SHIP inhibitors disclosed by the Kerr group are tryptamines with structures similar to K103 11. This compound was found to be a potent pan-SHIP1/2 inhibitor, and also was effective in causing apoptosis in breast cancer and multiple myeloma cell lines [19]. However, mice did not tolerate K103 11 very well, and therefore synthetic studies were performed to find an analog that was less problematic in this regard. This led to the discovery of the related K149 12, a pan-SHIP1/2 inhibitor which also showed promising activity against a number of colorectal cancer cell lines [102]. The results with the tryptamine and the quinoline pan-SHIP1/2 inhibitors provide good evidence that SHIP inhibition may be utilized as a molecular mechanism to treat a number of cancer types, and more work in this area appears warranted given the need for new cancer therapeutics with novel molecular mechanisms, and particularly those that also target the PI3K-AKT pathway essential for survival of virtually all forms of cancer.

A summary of the known SHIP inhibitors and associated biological data is shown in Table 1 below. As discussed above, a variety of structures have been shown to inhibit SHIP, and many have shown excellent activity in both in vitro assays and in cell lines. Some compounds have now advanced to animal models and ADME data on the structures has also recently become available. These small molecules should prove useful as tools for exploring the effects of SHIP modulation in cancer and other indications, and potentially even tested in a clinical setting.

## 9. SHIP Agonists

**Figure 8:**
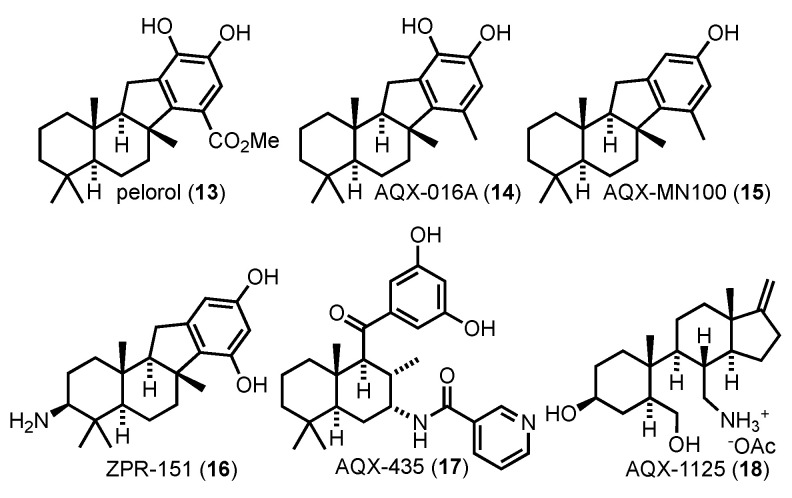
SHIP1 Agonists.

Early reports that SHIP is an allosterically modulated enzyme and that agonists have cytotoxic effects [145] have spurred the search for SHIP1 agonists. A number of SHIP1 agonists were identified by Andersen and co-workers, who screened the extracts of marine invertebrates for activity. These included the meroterpenoid pelorol (13), which was isolated from an extract of the Great Barrier Reef marine sponge *Dacylospongia elegans* collected in Papua New Guinea [177]. Pelorol induced a 2-fold activation of SHIP when tested at a concentration of 5 µg/mL [177]. A medicinal chemistry program was initiated and several analogs were synthesized and tested for SHIP activity [145,178,179]. Two of the analogs synthesized, AQX-016A (14) and AQX-MN100 (15) (Figure 8), exhibited higher levels of SHIP agonist activity [145]. AQX-016A, a catechol containing pelorol derivative, exhibited a 6-fold increase in the rate of phosphate hydrolysis for SHIP1 at 5 μg/mL. In addition to the higher activity, AQX-016A also showed selectivity for SHIP1 over SHIP2 by a factor of 5. However, one of the drawbacks of AQX-016A was the catechol functionality. Catechols are known to bind metals and can readily be oxidized to orthoquinones, which allow for protein modifications via Michael reactions. In order to mitigate these possible side effects AQX-MN100, the monophenolic version of AQX-016A was synthesized. AQX-MN100 possessed the same 6-fold activation of SHIP1, as well as maintaining the SHIP1 selectivity exhibited by that of AQX-016A [145]. This work provided strong proof of principle that SHIP1 agonists could be useful in vivo, and led to the founding of Aquinox Pharmaceuticals (Vancouver, Canada). One of the drawbacks of the terpenoid scaffold was the generally poor water solubility of the pelorol analogs. This was addressed by adding basic nitrogens to the scaffold with the synthesis of 16 and 17, which significantly increased water solubility and oral bioavailability [148,178,180]. AQX-435 was recently shown to significantly reduce tumor volume in murine models using a panel of mice bearing TMD8 or DLBCL PDX tumors [180]. These effects were shown to be synergistic with ibrutinib, and were consistent with a reduction in signaling through the PI3K pathway.

Further screening of small molecule libraries that had structures similar to pelorol led to the identification of AQX-1125 (18) [179], originally synthesized as an analog of the anti-inflammatory steroid IPL-576,092 [181]. AQX-1125 shows strong anti-inflammatory effects and excellent pharmacodynamics, with an approximate 20% increase in SHIP1 activation at a concentration of 300 μM [182,183]. Aquinox Pharmaceuticals have sponsored clinical trials on AQX-1125, making it the first small molecule to target SHIP1 that has proceeded to the clinic [184]. Unfortunately, AQX-1125 did not show significant efficacy to continue development as a treatment for interstitial cystitis (IC) and bladder pain syndrome (BPS), and its development has been discontinued. More recent studies have questioned whether AQX-1125 actually binds to SHIP1, indicating the molecule may be acting through other mechanisms [148].

A summary of the most utilized SHIP1 agonists is shown below in Table 2. The most well developed and characterized SHIP1 agonist has been AQX-1125 18, but the recent disclosure that AQX-1125 only weakly binds to SHIP1 [148] will likely result in a reinvigoration of research in this field, as the poor clinical results of AQX-1125 may be explained by the lack of binding. Further studies on the pelorol agonists ZPR-151 16 and AQX-435 17 would seem to be a logical starting point for this area. Pre-clinical studies clearly indicate a link between SHIP modulation and the growth and development of many cancers [185,186]. Armed with the structural information provided by x-ray crystallographers, medicinal chemistry studies can now be fully engaged to develop paralog selective potent inhibitors and agonists which can then be evaluated in tumor models. These compounds may also have uses in the deconvolution of the role of SHIP in PI3K signaling.

## 10. Potential Toxicities of SHIPi

Germline SHIP1 KO mice are born and weaned at the expected Mendelian frequency from matings of heterozygous parents [113]. However, after reaching adulthood they succumb to two severe and life-threatening pathologies with null mice rarely living beyond 12 weeks of age. These life-threatening pathologies are pulmonary consolidation with alveolar macrophage build-up and crystalline pneumonia [86] and severe ileitis that closely resembles human Crohn’s Disease [189]. The cause of these two pathologies are a precipitous decline in CD4 and CD8 T cells in both the gut and lung with a concomitant infiltration of hyper-responsive neutrophils and macrophages culminating in severe tissue damage and consequently loss of pulmonary and gastrointestinal function [111,189]. As sustained activation of the T and NK cells may lead to their exhaustion or disarming, SHIP1^−/−^ mice may also be prone to infection and malignancy; however, their greatly reduced lifespan has not enabled this question to be tested. Mice can be rescued from these severe pathologies by either allogeneic bone marrow transplantation [113,114] or adoptive transfer of syngeneic splenocytes from WT donors [111]. SHIP1^−/−^ mice also suffer from severe osteoporosis that was thought initially to result from a hyper-active osteoclast compartment. However, analysis of conditional SHIP1 mutant mice showed that osteoporosis results from SHIP1 deficiency in the mesenchymal stem cell compartment that is defective for differentiation to bone-forming osteoblasts in the absence of SHIP1 expression and increased PI3K/AKT signaling that promotes a β-Catenin/Id2 transcriptional circuit [85]. Thus far, neither life-threatening toxicity has been observed in vivo with either 3AC or the pan-SHIP1/2 inhibitors K118 or K149. However, a reduction in bone mass and density was observed with extended 3AC treatment [85], but not with its pan-SHIP1/2 inhibitory aminosteroid analog K118 [190]. It remains unclear as to why K118 failed to cause bone mass and density reduction as it inhibits SHIP1 equally or better than 3AC.

Mutations in the SHIP2 gene (INPPL1) was found to be causative of the autosomal recessive skeletal disorder opsismodysplasia, due to altered of chondrocyte differentiation [191]. The SHIP2-selective inhibitor AS1949490 has been shown to have potent anti-diabetic properties with no apparent toxicities reported to date [147]. However, this molecule has poor water solubility and would be difficult to develop. Recently, more SHIP2 selective inhibitors have been discovered as metformin (a FDA approved drug used in Type 2 diabetes) was recently shown to inhibit SHIP2 as well as the aforementioned Crizotinib derivatives like 23, which facilitates the evaluation of SHIP2 inhibitors for new therapeutic uses [172,192]. These structures may provide information about the possibility of using SHIP2 inhibitors clinically to treat diabetes and perhaps SHIP2 dependent cancers as well.

## 11. Conclusions and Future Directions

SHIP1 and SHIP2 have a central role in cancer development and progression, thus targeting SHIP could be a potential therapeutic in cancer with multiple applications. The PI3K/AKT pathway has been a central target for selective small molecule inhibitors and their association with chemotherapy, although resistance to these inhibitors are emerging [193]. For this reason, different enzyme targets that contribute in the same pathway could be developed to reduce onset of resistance. Alternatively, PI3K inhibitors and SHIP inhibitors could be utilized synergistically. For example, a combination of PI3K inhibitors and pan-SHIP1/2 inhibitors could lower the concentration of both PI(3,4,5)P_3_ and P(3,4)P_2_ on the cell membrane leading to the death of the cancer cells addicted to the AKT signaling cascade.

SHIP inhibitors have shown great potential as both chemotherapeutics and immunotherapeutics. More selective inhibitors with greater potency (perhaps in the nM range) could be discovered from computational screening utilizing the now available structures of the SHIP active sites. The optimization of their pharmacokinetics/pharmacodynamics would also be useful as more defined dosing strategies could be explored in vivo. Additionally, any possible activity of the metabolites of these small molecules could be determined. 

A possible approach for use of SHIP inhibition in both hematologic and non-hematologic cancer is alternation between pan-SHIP1/2 and SHIP1 selective therapies to improve the anti-cancer response. Pan-SHIP1/2 inhibitors would first be employed to ablate the cancers more effectively and then SHIP1-selective inhibition after a period of recovery so that tumor-reactive cytotoxic T and NK cells can be activated and increased by SHIP1 selective inhibition to eliminate the residual cancer burden. 

Of great interest would be to explore how the selective SHIP1 inhibitor 3AC boosts tumor immunity through T cells. Are only CD4^+^, CD8^+^ or both T cell types required for 3AC-induced tumor immunity and are these cells qualitatively changed by SHIPi to become more potent effector/memory cells or are their numbers simply increased by promoting their differentiation to T effector cells with enhanced survival in the tumor microenvironment. Moreover, 3AC treatment in the setting of long term survival could be better defined in order to understand how T and/or NK cell memory induction by SHIPi contributes to observed long-term protection from tumor relapse [118].

In order to reduce possible resistance to SHIPi, pan-SHIP1/2 inhibitors could also be combined with traditional chemotherapies to improve their cytotoxicity on cancer cells or chemotherapy could be followed by SHIP1 selective inhibitors, as immunotherapeutics, for an improved response to the tumor. Due to the minimal toxicity of SHIPi on healthy cells, it may be possible to develop combined therapies that are well tolerated even in cancer patients. SHIP inhibitors might even help with blood cell recovery through G-CSF induction [88] if given after radiation or chemotherapies that cause leukopenia. Other potential uses of SHIPi could be combining them with Immune Checkpoint Inhibitors (ICI) and Chimeric Antigen Receptor T cells (CAR-T) therapies. In the latter case to enhance the engraftment, persistence and function of the CAR-T or CAR-NK cell graft. Overall given the need for new methods to treat cancer and the interesting activity that has been demonstrated by SHIP inhibitors, it seems timely to now explore these possibilities further in earnest.

## Figures and Tables

**Table 1:** Summary of SHIP Antagonists.

Inhibitor	In Vitro (SHIP1)	In Vitro (SHIP2)	Cell Lines	Murine Data	ADME
1	Y (IC_50_ = 13 µM [147])	Y (IC_50_ = 0.62 µM [147])	Y [147]	Y [147]	Y [172]
2	Y (IC_50_ = 21 µM [163])	Y (IC_50_ = 0.57 µM [163])	Y [163]	-	-
3	-	Y (IC_50_ = 3.2 µM [172])	-	-	-
4	-	Y (IC_50_ = 2.0 µM [172])	Y [172]	Y [172]	Y [172]
5	Y (IC_50_ = 10 µM) [61]	-	Y [19,61]	Y [19,61]	-
6	Y (IC_50_ = 16 µM) [84]	Y (IC_50_ = 25 µM) [84]	Y [84]	-	-
7	Y (IC_50_ = 18 µM) [84]	Y (IC_50_ = 30 µM) [84]		-	-
8	Y (IC_50_ = 6 µM) [84]	Y (IC_50_ = 5-10 µM) [84]	Y [84]	Y [84]	Y [84]
9	Y [19]	Y [19]	Y [19]	-	-
10	Y [19]	Y [19]	Y [19]	-	-
11	Y [19]	Y [19]	Y [19]	-	-
12	Y (IC_50_ = 20-30 µM) [19]	Y (IC_50_ = 30 µM) [19]	Y [19]	-	-

**Table 2:** Summary of SHIP1 Agonists.

Agonist	In Vitro (SHIP1)	Cell Lines	Murine Data	ADME
13	Y [145,177]	-	-	-
14	Y [145]	Y [145]	Y [145]	-
15	Y [145]	Y [145]	Y [145]	-
16	Y [187]	-	-	-
17	Y [177,180]	Y [180]	Y [180]	-
18	Y [188]	Y [188]	Y [188]	Y [178]

## Data Availability

No new data was generated. Figures and tables are derived from resources in the references.
